# TGFBI Inhibits the Pyroptosis of Macrophages to Ameliorate Septic Shock

**DOI:** 10.1111/jcmm.70802

**Published:** 2025-10-13

**Authors:** Suzhao Zou, Lan Li, Bo Lv

**Affiliations:** ^1^ Department of Critical Care Medicine First Affiliated Hospital of Guizhou University of Traditional Chinese Medicine Guiyang China

**Keywords:** histone methylation, macrophage, pyroptosis, septic shock, TGFBI

## Abstract

Septic shock is one of the leading causes of morbidity and mortality in hospital patients. The present study aimed to investigate the potential of transforming growth factor β induced (TGFBI) in macrophage functions and progression of septic shock. Mice were treated with caecal ligation and puncture (CLP) to establish a septic shock model in vivo. Histopathologic analysis was performed using haematoxylin and eosin (HE) staining. Gene expression was detected using reverse transcription‐quantitative PCR and Western blot. Cytokine release was detected using an enzyme‐linked immunosorbent assay. The enrichment of TGFBI was detected using chromatin immunoprecipitation assay. Cellular functions were detected using Cell Counting Kit‐8, lactate dehydrogenase, flow cytometry, PI staining, and terminal deoxynucleotidyl transferase‐mediated dUTP nick end labelling staining assays. TGFBI was downregulated in septic shock patients and models. TGFBI overexpression suppressed the pyroptosis of macrophages by inhibiting the non‐canonical inflammasome, promoting the bacterial killing ability of macrophages. Wedelolactone‐mediated inhibition of pyroptosis alleviated sepsis‐induced multiple organ failure. Moreover, TGFBI inhibited M1 macrophage polarisation. Suppressor of variegation 3–9 homologue 2 (SUV39H2)‐mediated histone methylation of TGFBI, resulting in the downregulation of TGFBI. Signal transducer and activator of transcription 1 (Stat1) was identified as a new co‐activator of SUV39H2 to inhibit the transcription of TGFBI. However, inhibition of SUV39H2 and Stat1 upregulated TGFBI. Furthermore, Stat1 deficiency inhibited the pyroptosis of macrophages and alleviated sepsis‐induced multiple organ failure. In summary, our findings establish an immune checkpoint, whereby TGFBI, via inhibiting macrophage pyroptosis and M1 polarisation, suppresses the progression of septic shock.

## 1 Introduction

Sepsis shock is one of the leading causes of mortality in hospital patients, which is induced by pathogen infection and dysregulated host defence [1]. The clinical manifestations are inflammation‐related injury and organ dysfunction [2, 3]. The annual incidence of sepsis is 18 million, and the fatality rate is as high as 30%–70% [4]. The present strategies for sepsis are symptomatic treatments, including intravenous fluid administration and organ function support, while most aetiological therapies have failed [5, 6]. Therefore, developing a novel strategy for sepsis shock is urgently needed.

Macrophages are the intermediary bridging innate immunity and adaptive immunity [7]. In the initiation of sepsis, macrophages phagocytose pathogens, exerting protective functions [8, 9]. However, as sepsis progresses, the loss of macrophages exacerbates the development of sepsis [10]. Previous studies have demonstrated that the death of macrophages is present in the process of sepsis and related to the mortality of patients with sepsis [11, 12]. However, the underlying mechanisms are still unclear transforming growth factor β induced (TGFBI) is located in the extracellular matrix and is regulated by TGF‐β [13]. Low levels of TGFBI are associated with the pathogenesis of various inflammatory diseases, such as non‐asthmatic eosinophilic bronchitis and Sjögren syndrome [14, 15]. Moreover, TGFBI can be a biomarker for sepsis. Low levels of TGFBI are associated with poor outcomes of sepsis patients [16]. Interestingly, overexpressed TGFBI inhibits M1 macrophage polarisation and the inflammatory response [17]. Nonetheless, the mechanism is still unclear.

The study explored the potentials of TGFBI in septic shock. The potentials of TGFBI in macrophage pyroptosis may provide a novel insight into the immune system during the progression of septic shock.

## 2 Materials and Methods

### 2.1 Specimen

Blood samples were collected from paediatric diabetes‐related heart failure (HF) patients (*n* = 62) and healthy controls (*n* = 23) at the Second Affiliated Hospital of Guizhou University of Traditional Chinese Medicine. This study was approved and supervised by the Ethical Committee of the Second Affiliated Hospital of Guizhou University of Traditional Chinese Medicine. All the guardians have provided informed consent.

### 2.2 Animal Care

C57BL/6 mice (male, 4 weeks, 18–22 g) were purchased from the Experimental Animal Institution of Guizhou University of Traditional Chinese Medicine. Mice were housed in a 12‐h light/12‐h dark cycle at 22°C–24°C, with free access to food and water. This study was approved by the Animal Care Abroad of The Second Affiliated Hospital of Guizhou University of Traditional Chinese Medicine.

### 2.3 Caecal Ligation and Puncture (CLP)‐induced Septic Shock Model

CLP surgery was used to establish a sepsis mouse model. Briefly, mice were anaesthetised. The cecum was exposed and ligated. Then the cecum was punctured twice using an 18G needle. Afterwards, the peritoneum was closed and the mice were subcutaneously injected with 1 mL sterile saline. Afterwards, the mice were intraperitoneally injected with 20 mg/kg lipopolysaccharide to reproduce the murine endotoxemia. For lentivirus, the lentivirus plasmid carrying the full‐length TGFBI coding sequence and the lentivirus null control plasmids were constructed by GenePharm (Shanghai, China). Lv‐TGFBI or lv‐nc was intramyocardially injected at a dose of 1 × 10^9^/mouse. Lv‐nc is the lentivirus containing an empty macrophage CD68 promoter and serves as the control. For pyroptosis inhibition, Wedelolactone (WED) (HY‐N0551; MCE, USA) was intramyocardially injected at 20 mg/kg. The criteria used were referred to as previously described [18]. Mice, with three or more mentioned symptoms, were sacrificed.

### 2.4 Histopathologic Assessment

The heart or the aorta tissues were fixed in 4% paraformaldehyde (PFA) overnight, dehydrated in graded ethanol, cleared in xylene, embedded in paraffin, and cut into 20‐μm‐thick sections. After deparaffinisation, the sections were stained with haematoxylin for 8 min and eosin for 1 min, washed, dehydrated, and mounted. Three images of different views were taken blindly and three serial heart sections (1000 μM between them) were included for each heart.

### 2.5 Terminal Deoxynucleotidyl Transferase‐Mediated dUTP Nick End Labelling (TUNEL) Staining Assay

Tissues were immersed in PF *A. Coronal* brain sections and cells were washed in phosphate‐buffered saline with Tween (PBST) three times, respectively. The sections were blocked with 4% bovine serum albumin. Then the sections or cells were cultured with a TUNEL Assay kit (Takara, Japan). The images were captured using a Nikon microscope (CSU‐W1; Nikon Corporation, Tokyo, Japan). 4′,6‐diamidino‐2‐phenylindole (DAPI) counterstaining was used to visualise the nuclei. The images were captured using a fluorescence microscope (ECLIPSE Ts2; Nikon, Japan).

### 2.6 Preparation of Lentivirus (lv) Particles

LV particles were provided by GenePharma (Shanghai, China). TGFBI overexpression plasmids and the control (nc) were synthesised and cloned into the LV (titre for lv particles containing TGFBI or nc overexpression plasmids and vector: 1 × 10^13^ vg/mL).

### 2.7 Cell Culture

Human macrophage cell line THP1 (SNL‐044) was provided by Suncell, Wuhan, China. Cells were cultured in Dulbecco's Modified Eagle's Medium (DMEM) (12,491,015; Gibco, USA) supplemented with 10% fetal bovine serum (FBS) in 5% CO_2_ at 37°C. THP1 cells were stimulated with LPS (L2880‐10MG; Sigma‐Aldrich, Germany).

Cells were cultured in DMEM medium containing 10% FBS at 37°C in an air of 5% CO_2_. Then, cells were cultured with LPS + adenosine triphosphate (ATP).

For inhibition of pyroptosis, cells were treated with NLR family pyrin domain containing 3 (NLRP3) inhibitor (MCC950) (HY‐12815; MCE, USA).

For bacterial infection, cells were cultured with *Pseudomonas aeruginosa* (*P. ae*) strain (19660) and *Escherichia coli* ( *E. coli* ) strain (25922) at a multiplicity of infection of 20 based on LPS + ATP treatment (ATCC, USA).

### 2.8 Transfection

THP1 cells were transfected with TGFBI overexpression plasmids, empty vector, siRNA of TGFBI, suppressor of variegation 3–9 homologue 2 (SUV39H2) and signal transducer and activator of transcription 1 (Stat1), or the unspecified siRNA (USi) using Lipofectamine 2000 (11668019; Invitrogen, USA) (Table S1).

### 2.9 Enzyme‐Linked Immunosorbent Assay

The release of cytokines was determined using commercial kits (Abcam, UK).

### 2.10 Reverse Transcription‐Quantitative PCR (RT‐qPCR)

Total RNA was collected from cells and blood samples. cDNA synthesis was conducted using a SuperScript IV Kit (11483188001; Roche, Germany). PCR was performed using a Transcriptor One‐Stem RT‐PCR Kit (4655877001; Roche, Germany). Glyceraldehyde‐3‐phosphate dehydrogenase was used as an internal control. Relative RNA levels were detected using the 2^−ΔΔCq^ method (Table S2).

### 2.11 Western Blot

Total protein was collected from tissues. Protein concentration was calculated using bicinchoninic acid kits (ab102536; Abcam, UK). Protein (30 μL) was separated with 12% sodium dodecyl sulfate–polyacrylamide gel electrophoresis. Protein was moved to polyvinylidene fluoride membranes sealed with 5% skimmed milk. Afterwards, the membranes were incubated with primary antibodies, such as anti‐TGFBI (ab170874; 1: 1000, Abcam, UK), anti‐NLRP3 (ab263899; 1: 1000, Abcam, UK), anti‐pro‐caspase1 (casp1) (ab179515; 1: 1000, Abcam, UK), anti‐pro‐casp11 (ab246496; 1: 1000, Abcam, UK), anti‐gasdermin‐N terminal domain (GSDMD‐N) (ab215203; 1: 1000, Abcam, UK), anti‐SUV39H2 (ab190870; 1: 1000, Abcam, UK), anti‐lysine demethylase 3B (KDM3B) (ab271046; 1: 1000, Abcam, UK), anti‐H3K9me3 (ab176916; 1: 1000, Abcam, UK), anti‐β‐actin (ab8227; 1: 1000, Abcam, UK) and anti‐H3 (ab1791; 1: 1000, Abcam, UK), and then with secondary antibody (ab6721; 1: 5000, Abcam, UK). Finally, the band was pictured with an enhanced chemiluminescence kit (6104‐58‐1; Sigma‐Aldrich, Germany).

### 2.12 Chromatin Immunoprecipitation (ChIP) Assay

An EpiQuik Chromatin Immunoprecipitation Assay Kit (P‐2002‐3; EpiGentek, Brooklyn, NY) was used according to the manufacturer's instructions. Ten micrograms of anti‐H3K9me2 antibody (ab1220; 1: 50, Abcam, UK) or control IgG antibody (ab172730; 1: 50, Abcam, UK) were used for immunoprecipitation. Next, RT‐qPCR was applied to detect the enrichment of DNA fragments in the predicted SUV39H2 binding sites.

### 2.13 Cell Counting Kit‐8 (CCK‐8) Assay

Cells were seeded into a 24‐well plate. Then CCK‐8 solution (C0038; Beyotime, China) was added and the mixture was cultured for 4 h. Finally, the results were detected using a microplate reader at the wavelength of 450 nm.

### 2.14 Flow Cytometry

For detecting macrophage, cells were digested, lysed, and filtered. Afterwards, cells were resuspended and incubated with mouse Fc receptor blocker and then with primary antibodies in shade, such as anti‐CD86 (ab239075; 1: 500, Abcam, UK), anti‐TNF‐α (ab300093; 1: 50, Abcam, UK) and stained with intracellular antibodies. The results were analysed using flow cytometry (BD Biosciences, USA).

### 2.15 Bioinformatics Analysis

GSE131761 was applied to determine the differentially expressed genes (DEGs) after transfection in septic shock patients.

### 2.16 Statistical Analysis

All data were analysed using Graphpad 9.5.1 and presented as mean ± SD. The difference was evaluated using Student's t test and ANOVA followed by Bonferroni post hoc test. Diagnostic values of DUSP5 were analysed using a receiver operating characteristic (ROC) curve. *p* < 0.05 indicated statistical significance.

## 3 Results

### 3.1 Characteristics of Septic Shock Patients

As shown in Table S3, compared with healthy volunteers, the total leukocyte count was significantly increased in septic shock patients, whereas lymphocyte cell count was reduced. Moreover, the levels of brain natriuretic peptide (BNP), C‐type reactive protein (CRP) and procalcitonin (PCT) were significantly increased in septic shock patients. However, the comparison in age and gender between the two groups showed no significant difference.

### 3.2 TGFBI Is Downregulated in Septic Shock Patients

**FIGURE 1:**
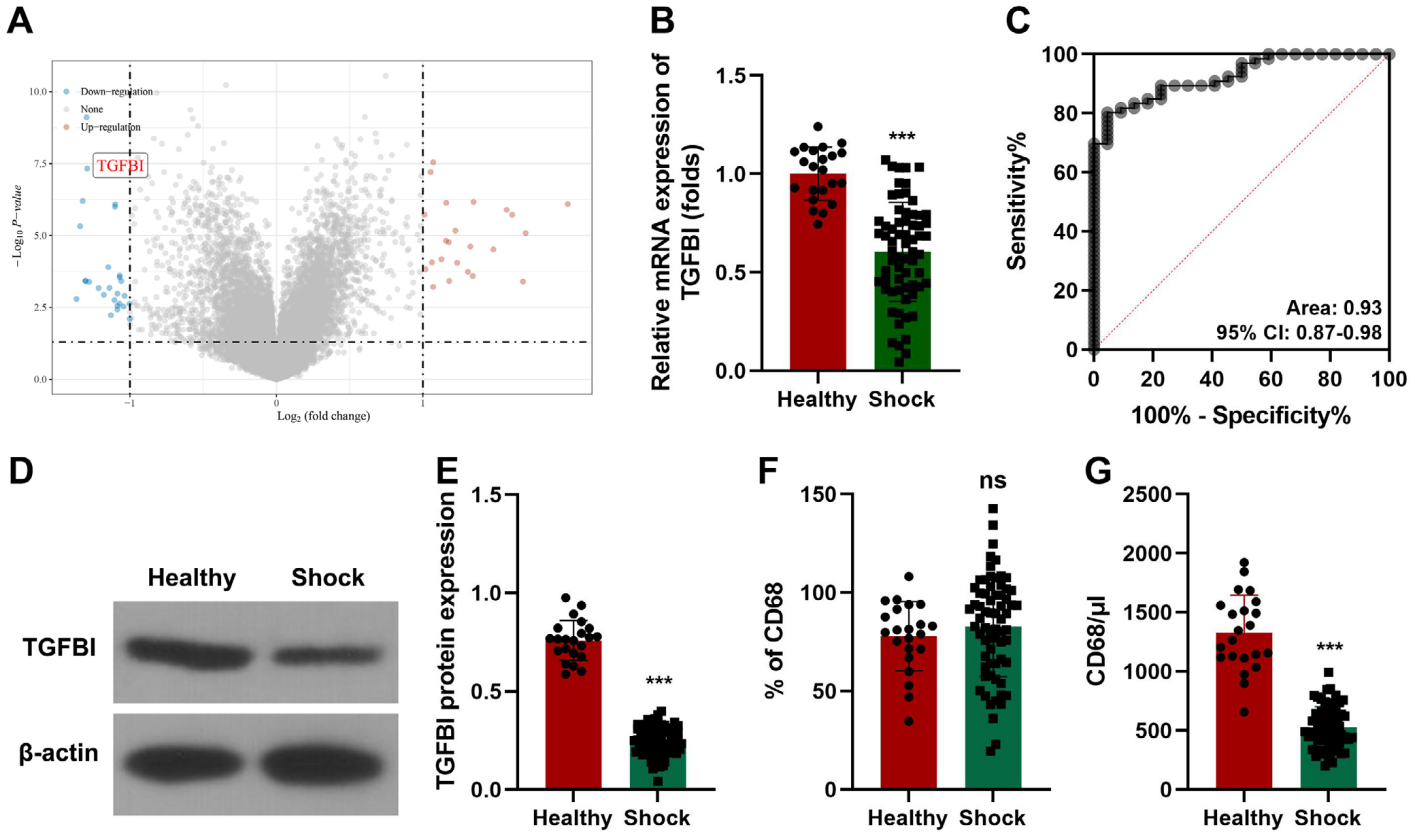
TGFBI expression in septic shock patients. (A) DEGs in septic patients was analysed using GSE131761. (B) TGFBI mRNA expression in septic patients was detected using RT‐qPCR. (C) ROC curve analysis of TGFBI mRNA expression in septic patients. (D, E) TGFBI protein expression in septic patients was detected using western blot. (F, G) The percentages and number of macrophages was detected using flow cytometry. ***p < 0.001.

GSE131761 was used to analyse the DEGs in septic shock patients (Figure 1A). Among the top 5 downregulated genes, TGFBI is reported to be abnormally expressed in sepsis [16, 17]. Therefore, TGFBI was chosen for further study. TGFBI was significantly downregulated in septic shock patients (Figure 1B). TGFBI mRNA expression can be a sensitive biomarker for septic shock patients (Figure 1C). TGFBI protein expression was also downregulated in septic shock patients (Figure 1D,E). TGFBI is predominantly secreted by macrophages. Therefore, we determined macrophages in PBMCs. We found that the percentages of macrophages showed no significant alteration (Figure 1F), whereas the number was significantly decreased in septic patients (Figure 1G).

### 3.3 TGFBI Improves Sepsis Outcomes

**FIGURE 2:**
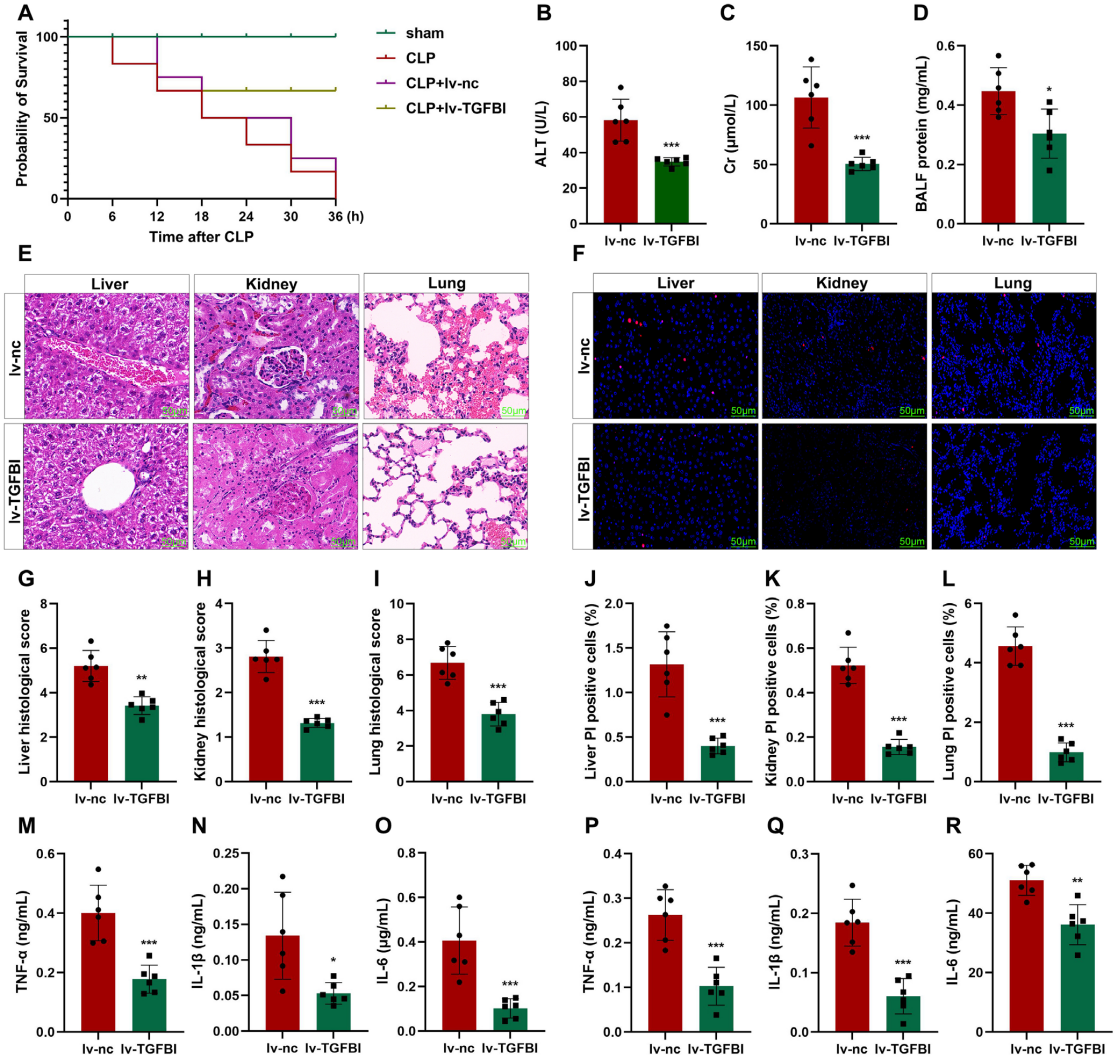
TGFBI improves sepsis outcomes. (A) The survival rates of mice were detected using Kaplan–Meier analysis. The levels of ALT (B), Cr (C), and total BALF (D). (E, G–I) Histological analysis was detected using HE staining. (F, J–L) Macrophage death was detected using TUNEL staining. (M–R) Cytokine release was detected using ELISA. *p < 0.05, **p < 0.01, ***p < 0.001.

To confirm the roles of TGFBI in septic shock, mice were injected with lv‐TGFBI. We found that CLP treatment significantly reduced the survival rates of mice (Figure 2A), which was alleviated by overexpressed TGFBI. Moreover, the increase in ALT, Cr, and total BALF induced by CLP treatment was significantly reversed by overexpressed TGFBI (Figure 2B–D). Histological analysis showed that CLP‐mediated severe multiple organ injury (Figure 2E,G–I) and cell death of macrophages (Figure 2F,J–L) were alleviated by overexpressed TGFBI. We also found that CLP treatment‐mediated filtration of M1 macrophages was alleviated by overexpressed TGFBI (Figure S1). To further illustrate the effect of TGFBI on inflammatory response in septic shock, we further detected the pro‐inflammatory cytokines in plasma and peritoneal lavage fluid (PLF). As shown in Figure 2M–R, both in plasma and PLF, overexpressed TGFBI significantly decreased the release of tumour necrosis factor α (TNF‐α), interleukin 1β (IL‐1β), and IL‐6, which are mainly secreted by macrophages. These results suggested that TGFBI alleviates susceptibility to infection‐induced multiple organ failure (MOF) through inhibiting M1 macrophage polarisation and death.

### 3.4 TGFBI Suppresses the Pyroptosis of Macrophages Through Non‐Canonical Inflammasome Blockade

**FIGURE 3:**
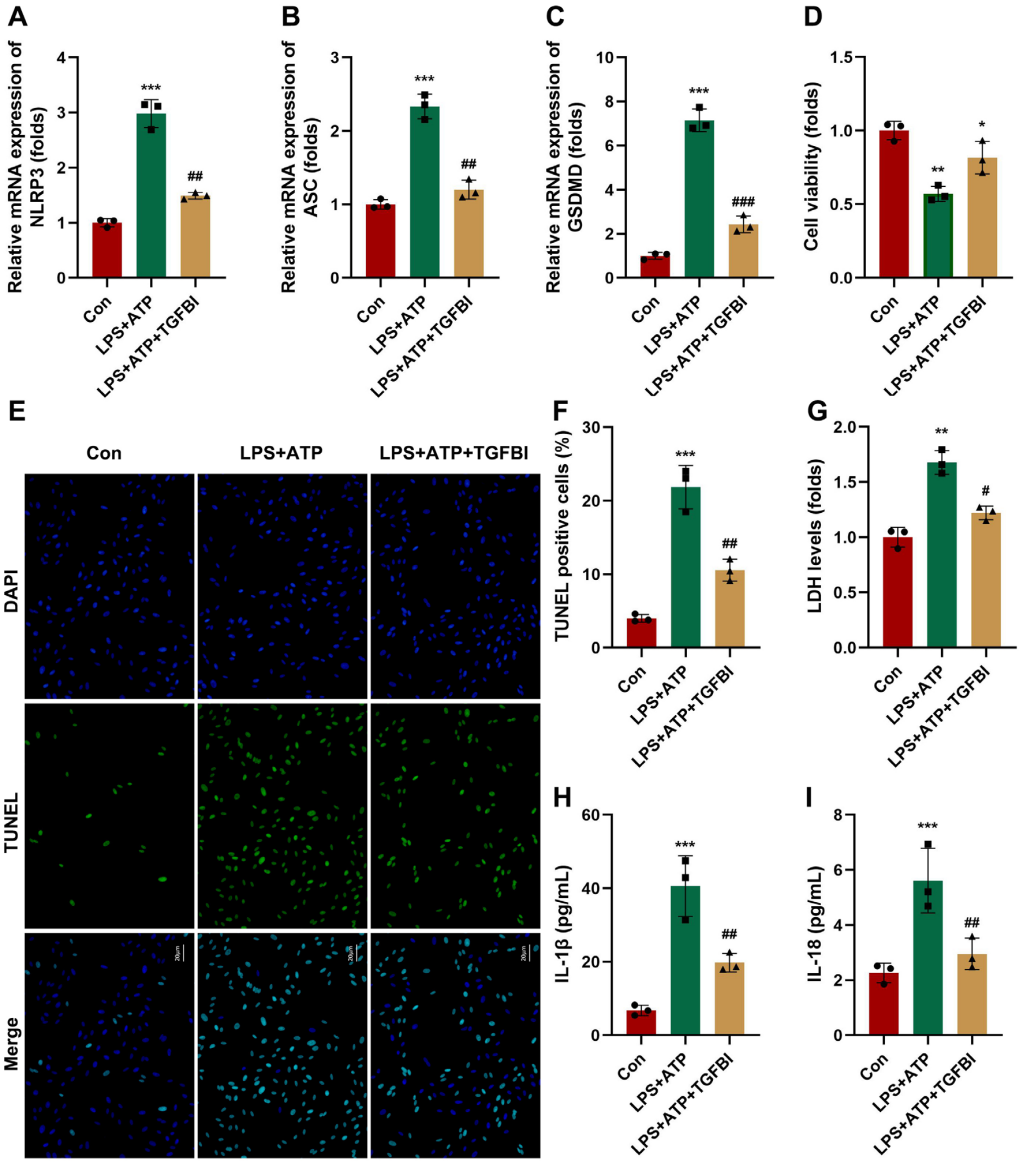
TGFBI suppresses the pyroptosis of macrophages. (A–C) mRNA expression in macrophages was detected using RT‐qPCR. (D) Cell viability was detected using CCK‐8 assay. (E, F) Macrophage death was detected using TUNEL assay. (G) Cytotoxicity was detected using LDH assay. (H, I) Cytokine release was detected using ELISA. *p < 0.05, **p < 0.01, ***p < 0.001, ^#^ *p* < 0.05, ^##^ *p* < 0.01.

The death of macrophages exacerbates the progression of sepsis [19, 20]. Therefore, we speculated whether TGFBI was associated with the inflammatory‐related cell death in sepsis. As shown in Figure 3A–C, overexpressed TGFBI significantly alleviated the effects of LPS + ATP and suppressed the mRNA expression of NLRP3, ASC, and GSDMD. Moreover, overexpressed TGFBI significantly increased the cell viability of macrophages (Figure 3D), as well as inhibited macrophage death (Figure 3E,F). We also found that overexpressed TGFBI significantly inhibited cytotoxicity in macrophages (Figure 3G). Additionally, overexpressed TGFBI significantly suppressed the release of IL‐1β and IL‐18 (Figure 3H,I). Furthermore, overexpressed TGFBI or inhibition of pyroptosis using the NLRP3 inhibitor promoted the bacterial killing ability of macrophages (Figure S2). These results suggested that TGFBI could suppress the pyroptosis of macrophages in sepsis.

**FIGURE 4:**
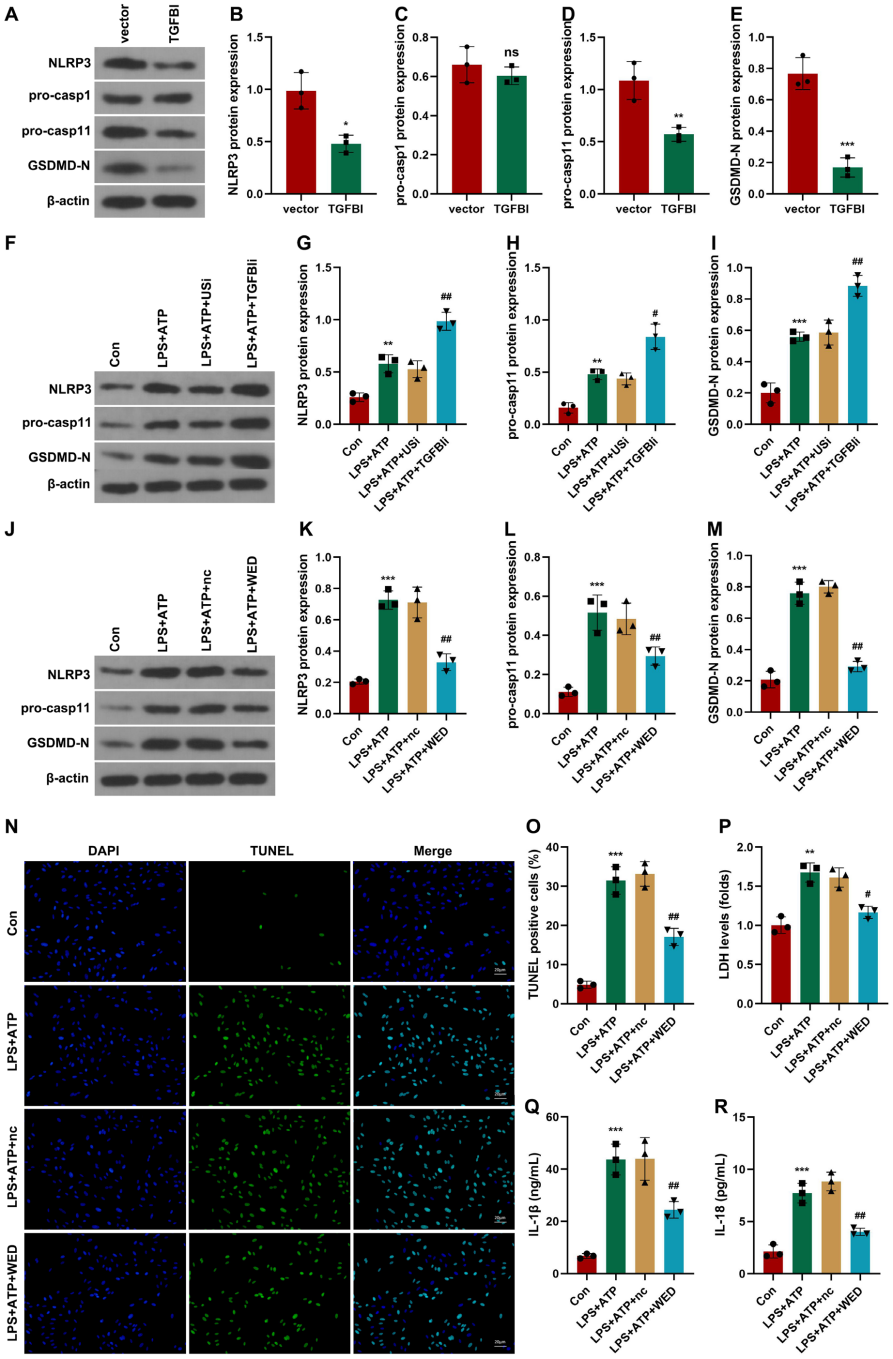
TGFBI suppresses the pyroptosis of macrophages though non‐canonical inflammasome blockade. (A–E) Protein expression in macrophages was detected using western blot after transfection with TGFBI overexpression plasmids. (F–I) Protein expression in macrophages was detected using western blot after transfection with TGFBIi. (J–M) Protein expression in macrophages was detected using western blot after treatment with WED. (N, O) Macrophage death was detected using TUNEL assay. (P) Cytotoxicity was detected using LDH assay. (Q, R) Cytokine release was detected using ELISA. *p < 0.05, **p < 0.01, ***p < 0.001, ^#^ *p* < 0.05, ^##^ *p* < 0.01.

The pyroptosis is regulated by canonical (caspase1) and non‐canonical (caspase11) pathways [21, 22]. To verify this, we determined the expression of caspase1 and caspase11. As shown in Figure 4A–E, overexpressed TGFBI significantly suppressed the protein expression of NLRP3, pro‐casp11, and GSDMD‐N, whereas it showed no significant changes in pro‐casp1 expression. However, TGFBI knockdown significantly enhanced the effects of LPS + ATP and increased the protein expression of NLRP3, pro‐casp11, and GSDMD‐N (Figure 4F–I). Treatment with the casp11 inhibitor (WED) significantly alleviated the effects of LPS + ATP and downregulated NLRP3, pro‐casp11, and GSDMD‐N (Figure 4J–M). Moreover, WED treatment significantly suppressed cell death induced by LPS + ATP treatment (Figure 4N,O) and cytotoxicity in macrophages induced by LPS + ATP treatment (Figure 4P). The LPS + ATP treatment‐induced increase in the release of IL‐1β and IL‐18 was also reversed by WED treatment (Figure 4Q,R).

Taken together, TGFBI suppresses the pyroptosis of macrophages through inhibiting the activation of the non‐canonical inflammasome.

### 3.5 WED Inhibits Septic Shock‐Induced Macrophage Pyroptosis

**FIGURE 5:**
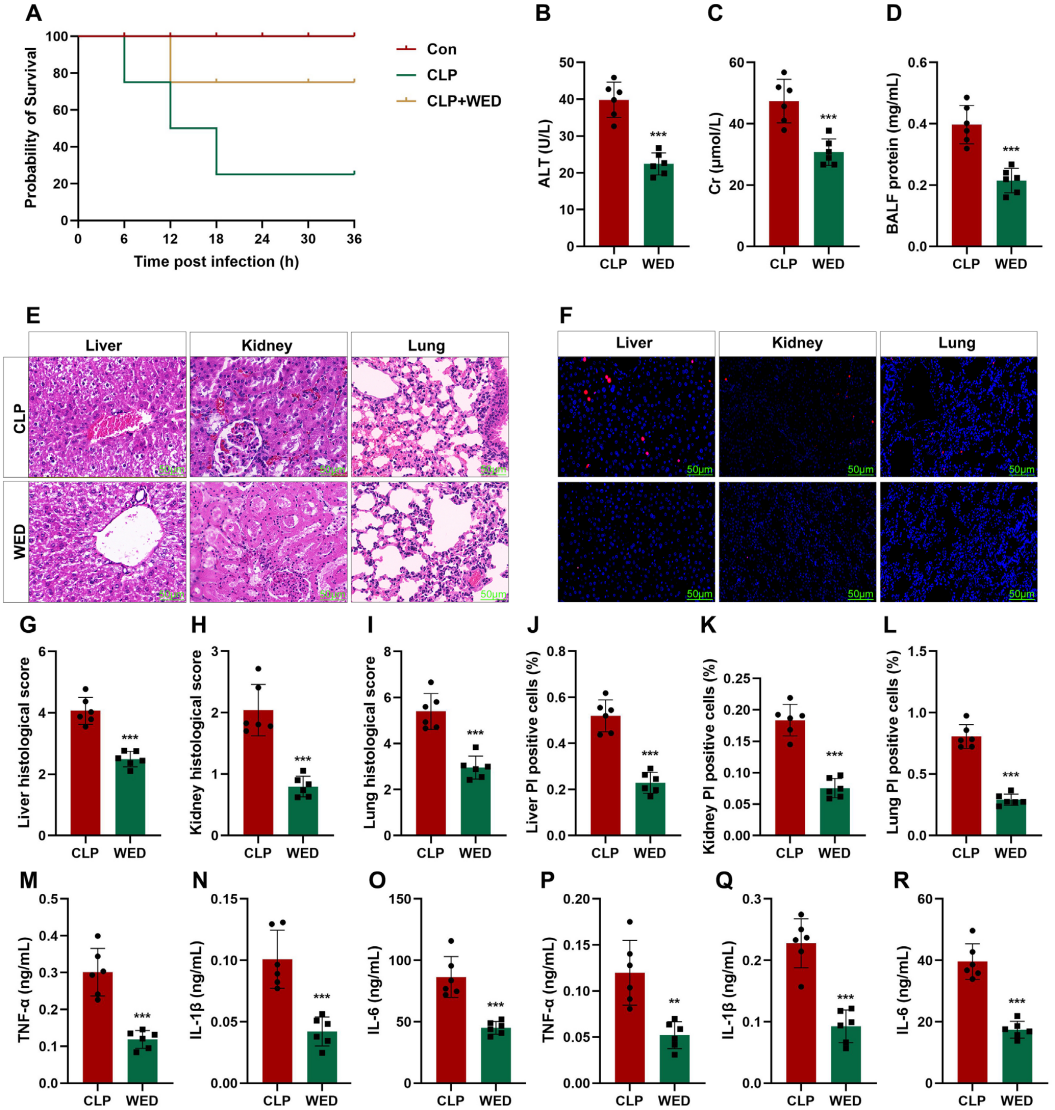
WED inhibits septic shock‐induced macrophage pyroptosis. (A) The survival rates of mice were detected using Kaplan–Meier analysis. The levels of ALT (B), Cr (C), and total BALF (D). (E, G–I) Histological analysis was detected using HE staining. (F, J–L) Macrophage death was detected using TUNEL staining. (M‐R) Cytokine release was detected using ELISA. **p < 0.01, ***p < 0.001.

To confirm the roles of macrophage pyroptosis in septic shock, mice were injected with WED. We found that WED significantly improved the survival rates of mice (Figure 5A). WED also significantly reduced the levels of ALT, Cr, and total BALF (Figure 5B–D). Moreover, WED alleviated severe multiple organ injury (Figure 5E,G–I) and the death of macrophages (Figure 5F,J–L). We also found that CLP treatment‐induced filtration of M1 macrophages was alleviated by WED (Figure S3). We also found that WED significantly decreased the release of TNF‐α, IL‐1β, and IL‐6 (Figure 5M–R). These results suggested that inhibition of macrophage pyroptosis reduced the susceptibility to infection‐induced MOF in septic shock.

### 3.6 SUV39H2 Downregulates TGFBI via Mediating Methylation of Histone H3K9me2

**FIGURE 6:**
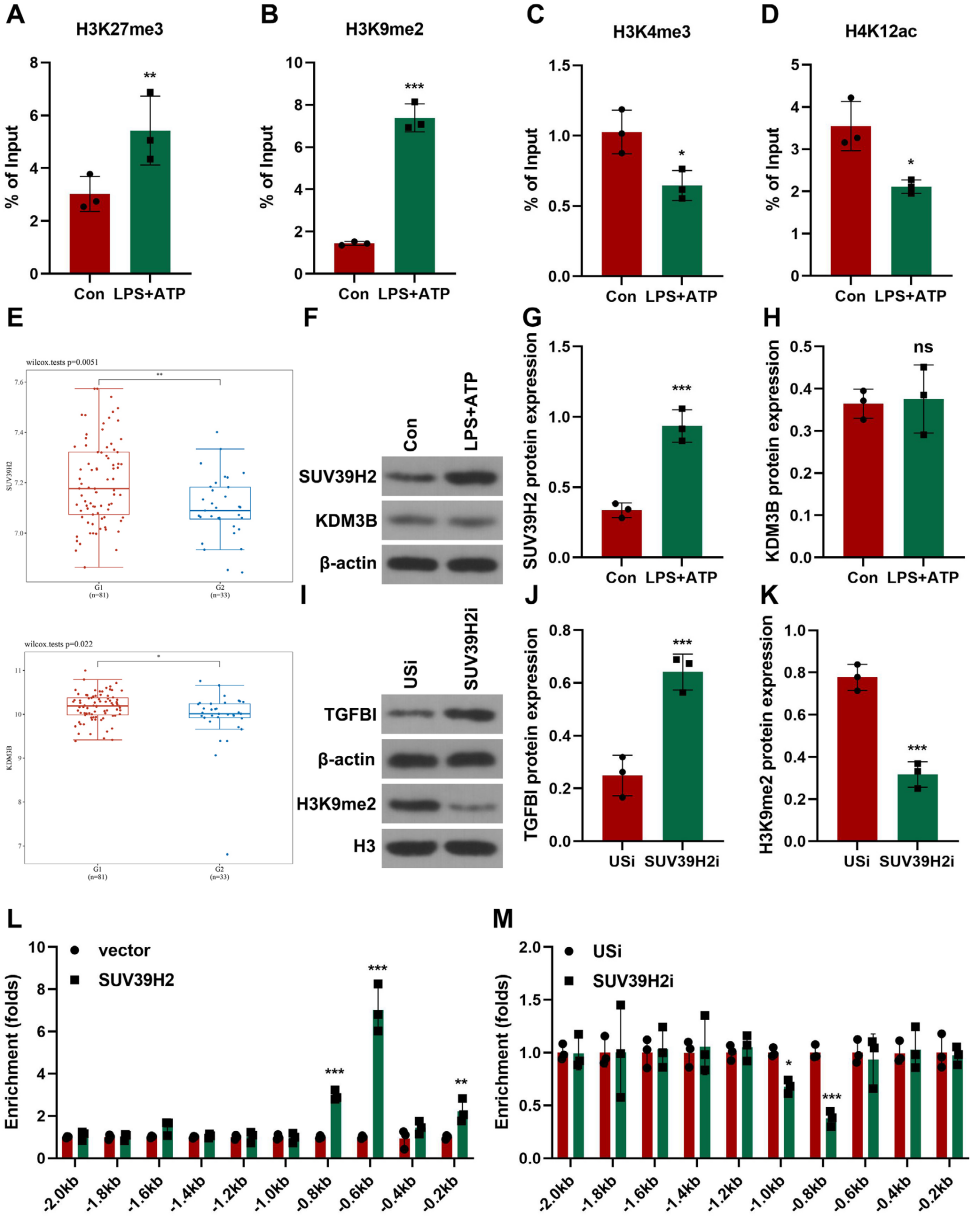
SUV39H2 downregulates TGFBI via mediating methylation of histone H3K9me2. (A–D) Global levels of H3K4me3, H3K9me2, H3K27me3, and H3K18la was detected using ChIP assay. (E) SUV39H2 and KDM3B expression was analysed using GSE131761. (F–H) Protein expression in macrophages was detected using western blot. (I–K) Protein expression in macrophages was detected using western blot. (L, M) The enrichment of H3K9me2 in the promoter of TGFBI was detected using ChIP assay. *p < 0.05, **p < 0.01, ***p < 0.001.

Accessible chromatin structure permits histone methylation of TGFBI [23]. H3K4me3, H3K9me2, H3K27me3, as well as H3K18la are involved in the pathogenesis of sepsis [24, 25, 26, 27, 28]. Therefore, we detected the alteration of H3K4me3, H3K9me2, H3K27me3, and H3K18la in macrophages after LPS + ATP treatment using ChIP PCR. The global levels of H3K27me3 and H3K9me2 were significantly increased (Figure 6A,B), whereas H3K4me3 and H3K18la were decreased (Figure 6C,D). Moreover, the change of H3K9me2 was more remarkable. Therefore, H3K9me2 was chosen for further study. To further confirm this, we analysed the expression of histone methyltransferases (HMTs) that methylate H3K9me2. We found that SUV39H2 and KDM3B were significantly upregulated (Figure 6E). To confirm this, we determined the protein expression of SUV39H2 and KDM3B. We found that LPS + ATP treatment significantly increased the protein expression of SUV39H2, but not KDM3B (Figure 6F–H). Therefore, SUV39H2 may be the histone methyltransferase that regulates the methylation of histone H3K9me2 in the promoter of TGFBI. Moreover, SUV39H2 knockdown significantly decreased the global levels of H3K9me2, but upregulated the protein expression of TGFBI (Figure 6I–K). ChIP PCR showed that SUV39H2 overexpression significantly increased the enrichment of H3K9me2 in the promoter of TGFBI (Figure 6L), whereas SUV39H2 knockdown exerted the opposite effects (Figure 6M). These findings suggested that SUV39H2‐mediated histone methylation of TGFBI downregulated the latter expression.

### 3.7 Stat1 Acts as a New Co‐Activator of SUV39H2

**FIGURE 7:**
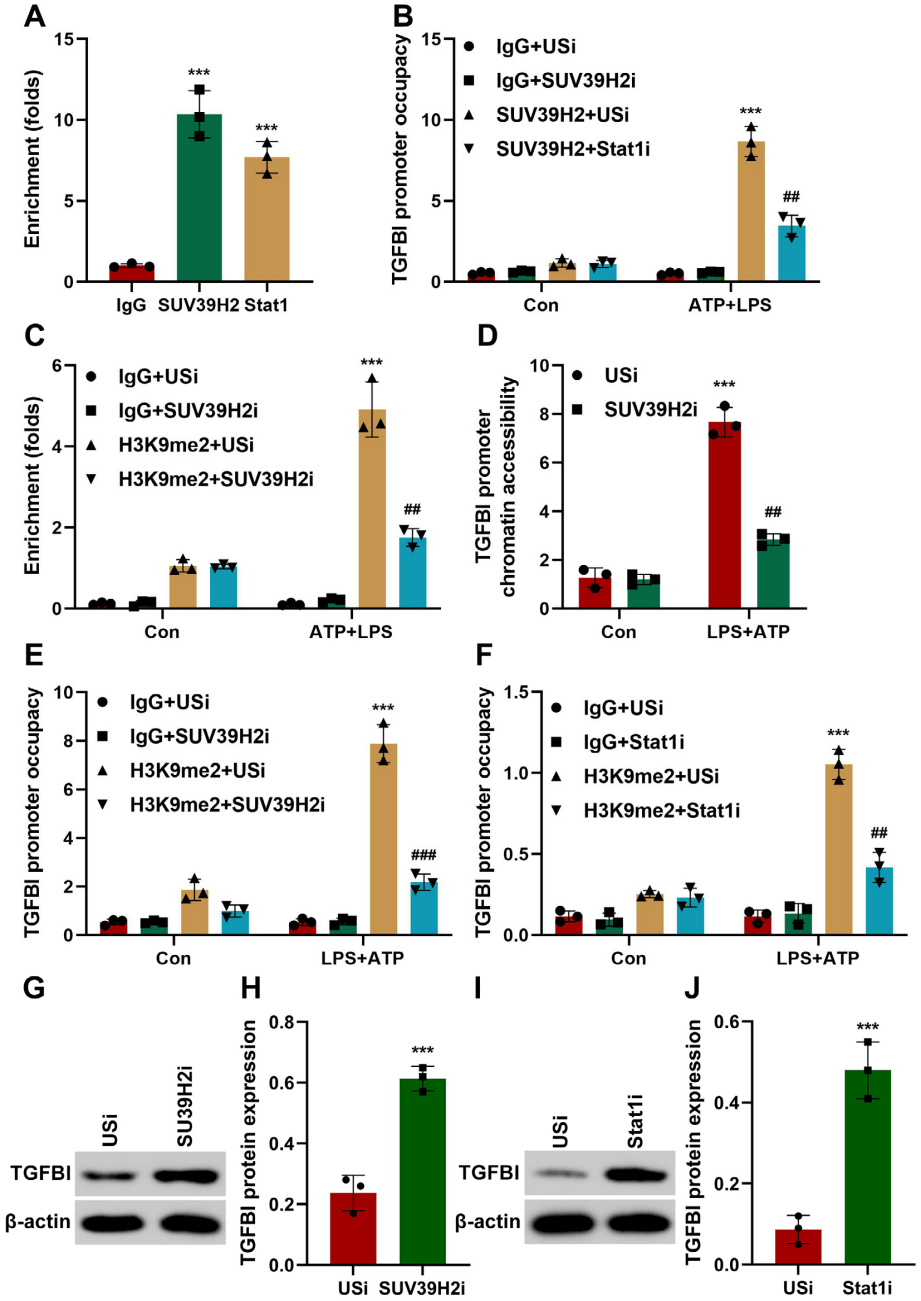
Stat1 acts as a new co‐activator of SUV39H2. (A) SUV39H2 and Stat1 ChIP recruitment at the ChoRE‐containing region of TGFBI promoter. (B, C) The recruitment of SUV39H2 to TGFBI promoter was detected using ChIP assay. (D–F) Chromatin accessibility and occupancy of THGBI promoter was detected using ChIP assay. (G–J) THGBI protein expression was detected using Western blot. ***p < 0.001, ^##^ *p* < 0.01, ^###^ *p* < 0.001.

Stat1 is a master regulator of M1 macrophage polarisation and pyroptosis [29, 30]. Moreover, histone methyltransferases and transcription factors frequently share regions in the promoter of the targets. Therefore, we hypothesised that Stat1 may interact with SUV39H2 to promote pyroptosis via regulating TGFBI expression. To confirm this, cells were co‐transfected with SUV39H2 and Stat1. We found that the co‐transfection significantly increased the protein expression of NLRP3, pro‐casp11, and GSDMD‐N (Figure S3). The results from ChIP PCR showed that SUV39H2 was co‐recruited with Stat1 in the promoter of TGFBI (Figure 7A). Moreover, after LPS + ATP treatment, SUV39H2 is recruited to the promoter of TGFBI, which was antagonised by Stat1 knockdown (Figure 7B). These results demonstrate that the binding of SUV39H2 on the ChoRE‐containing promoter is dependent on Stat1. However, SUV39H2 knockdown inhibited H3K9me2 methylation in the promoter of TGFBI (Figure 7C). Moreover, SUV39H2 knockdown reduced the chromatin promoter accessibility of TGFBI and the recruitment of Stat1 and RNA pol II in the promoter of TGFBI (Figure 7D–F). Moreover, Stat1 or SUV39H2 knockdown significantly downregulated the protein expression of TGFBI (Figure 7G–J). These results suggested that SUV39H2 interacts with Stat1 to regulate the transcription of TGFBI.

### 3.8 Stat1 Deficiency Inhibits Macrophage Pyroptosis

**FIGURE 8:**
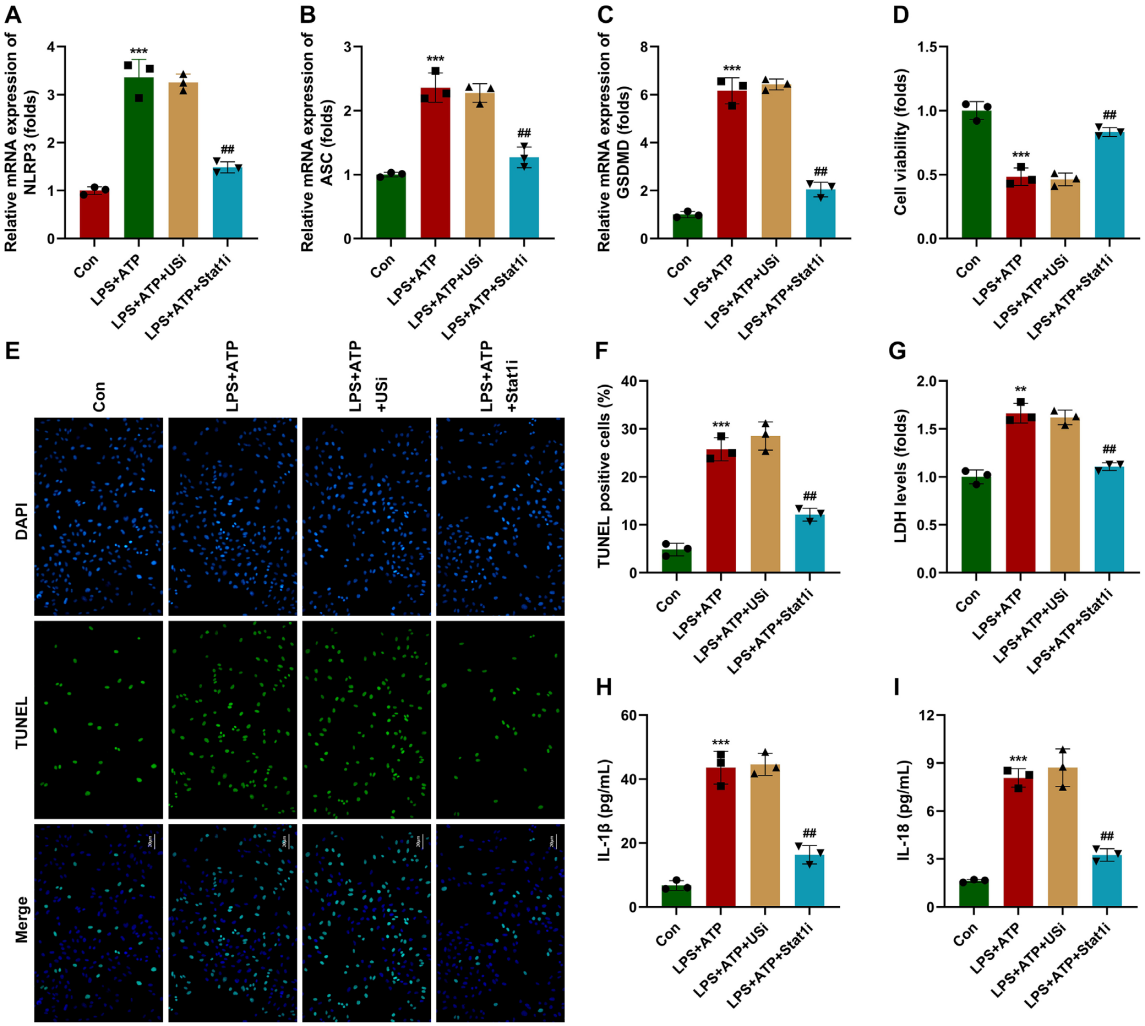
Stat1 deficiency inhibits macrophage pyroptosis in vitro. (A–C) mRNA expression in macrophages was detected using RT‐qPCR. (D) Cell viability was detected using CCK‐8 assay. (E, F) Macrophage death was detected using TUNEL assay. (G) Cytotoxicity was detected using LDH assay. (H, I) Cytokine release was detected using ELISA. **p < 0.01, ***p < 0.001, ^##^ *p* < 0.01.

Stat1 knockdown significantly suppressed the mRNA expression of NLRP3, ASC, and GSDMD compared with the LPS + ATP + lv‐USi group (Figure 8A–C). Moreover, Stat1 knockdown significantly suppressed the cell viability of macrophages (Figure 8D). Stat1 knockdown significantly alleviated the effects of LPS + ATP and suppressed the death (Figure 8E,F) as well as increased the cytotoxicity of macrophages (Figure 8G). Additionally, Stat1 knockdown significantly reduced the release of IL‐1β and IL‐18 (Figure 8H,I). These findings suggested that Stat1 knockdown alleviates the pyroptosis of macrophages in septic shock.

**FIGURE 9:**
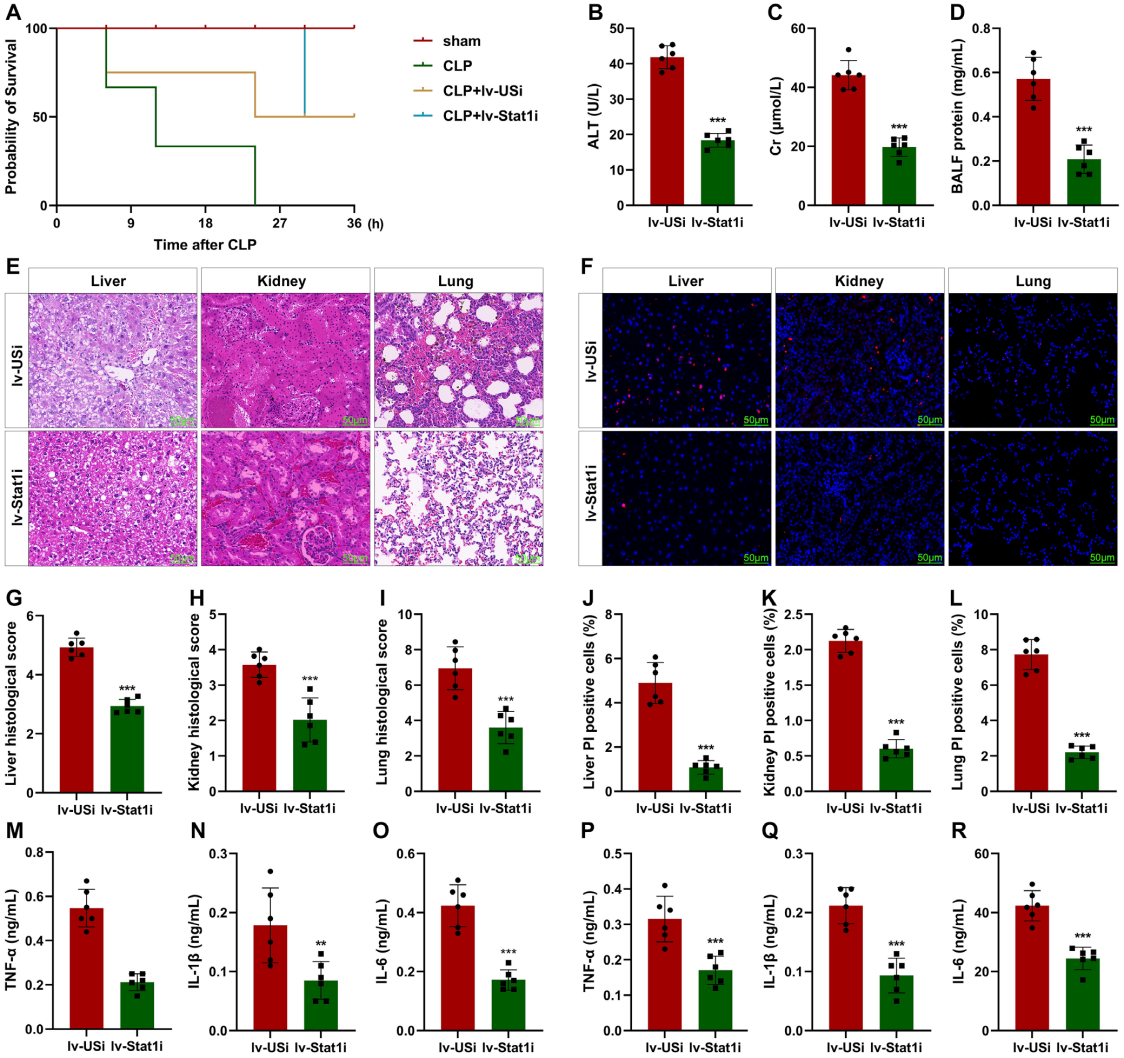
Stat1 deficiency inhibits macrophage pyroptosis in vivo. (A) The survival rates of mice were detected using Kaplan–Meier analysis. The levels of ALT (B), Cr (C), and total BALF (D). (E, G–I) Histological analysis was detected using HE staining. (F, J–L) Macrophage death was detected using TUNEL staining. (M–R) Cytokine release was detected using ELISA. **p < 0.01, ***p < 0.001.

To confirm this, mice were injected with lv‐Stat1i. We found that Stat1 knockdown significantly increased the survival of mice (Figure 9A). Stat1 knockdown significantly reduced the levels of ALT, Cr, and total BALF (Figure 9B–D). Stat1 knockdown alleviated severe multiple organ injury (Figure 9E,G–I) and the death of macrophages (Figure 9F,J–L). Stat1 knockdown significantly decreased the release of TNF‐α, IL‐1β, and IL‐6 in both plasma and PLF (Figure 5M–R). These results suggested that inhibition of Stat1 reduced susceptibility to infection‐induced MOF in septic shock.

## 4 Discussion

Sepsis is a progressive life‐threatening condition characterised by immune disorders and MOF [31]. Although immune cell death increases susceptibility to develop infectious diseases [32], the underlying mechanism remains unclear. The present study evidenced that the pyroptosis of macrophages led to immune dysfunction. TGFBI was downregulated in septic shock. However, overexpressed TGFBI suppressed macrophage pyroptosis and pro‐inflammatory M1 polarisation, and restored sepsis‐induced MOF. Moreover, SUV39H2 interacted with Stat1 to suppress the transcription of TGFBI, resulting in the activation of a non‐canonical inflammasome and subsequent pyroptosis of macrophages. Interestingly, pyroptosis inhibition suppressed the death of macrophages.

Continuous release of pro‐inflammatory mediators induces the recruitment of immune cells, which, in turn, further promotes the progression of sepsis‐induced inflammation via secreting pro‐inflammatory cytokines [33, 34]. As the progression of sepsis continues, excessive inflammatory response induces the damage of the resident as well as immune cells [35]. This excessive immune system activation and cascading inflammation contribute to immunosuppression and immunosenescence [6, 36]. For instance, activated neutrophils promote the apoptosis of T cells, leading to immunosuppression in sepsis [37]. IL‐33‐mediated senescence of naïve T cells impedes host defence in sepsis [38]. Macrophages bridge innate immunity and adaptive immunity [7]. However, the loss of macrophages exacerbates the progression of sepsis [10]. In the present study, the number of macrophages was reduced in septic shock patients, which is consistent with Wu et al.'s [39] study. The death of macrophages deteriorates septic inflammation as well as dampens bacterial killing ability and inhibits efferocytosis. To determine whether the loss of macrophages could stimulate dysregulation of host defence and induce MOF, mice were treated with CLP, and the macrophages were treated with WED. We found that sepsis‐induced inflammatory response and MOF were accompanied by the loss of macrophages. Moreover, inhibiting the pyroptosis of macrophages restored the bacterial killing ability of macrophages and alleviated MOF.

TGFBI is frequently downregulated in inflammatory diseases [15, 16]. However, overexpressed TGFBI maintains cartilage homeostasis and inhibits the pathogenesis of osteoarthritis [39]. Low levels of TGFBI are also associated with the progression of acute histological chorioamnionitis [40]. In the pathogenesis of sepsis, low levels of TGFBI predict poor prognosis and rapid disease progression [17]. Herein, TGFBI was downregulated in septic shock patients, which was accompanied by the loss of macrophages. Thence, we hypothesised that TGFBI deficiency may predict immunosuppression, which is characterised by the loss of immune cells [41]. To confirm this, mice were transiently expressed in mice after CLP treatment. Interestingly, overexpressed TGFBI suppressed the inflammatory response and the pyroptosis of macrophages, promoted the bacterial killing ability, and alleviated sepsis‐induced MOF. These findings suggested that TGFBI may play a protective role in septic shock.

SUV39H2 is a member of lysine methyltransferases [42]. The aberrantly expressed SUV39H2 is associated with the pathogenesis of various diseases, including cancer, cardiovascular diseases, and liver diseases [43, 44, 45]. However, the roles of SUV39H2 may vary with the types of diseases. For instance, SUV39H2 protects against oxidative stress‐induced cardiomyocyte senescence [46]. Overexpressed SUV39H2 promoted the aggressiveness of nasopharyngeal carcinoma [47]. Fan et al. [48] also demonstrate that SUV39H2‐mediated pro‐inflammatory M1 macrophage polarisation promotes the progression of nonalcoholic steatohepatitis. Therefore, to verify the roles of SUV39H2 in septic shock is essential. In this study, SUV39H2 was downregulated in septic shock patients. SUV39H2, as a key regulator that catalyses the formation of H3K9 dimethylation (H2K9me2), is involved in regulating biological processes via mediating the methylation of H3K9 in the promoter of the targets [49]. For example, SUV39H2 enhances the metastasis of colorectal cancer via histone methylation of the slit guidance ligand 1 promoter [50]. Moreover, SUV39H2 promotes the progression of intervertebral disc degeneration via driving the lysine methylation of protein phosphatase 1 catalytic subunit alpha [51]. In this study, SUV39H2 mediated H3K9 dimethylation of TGFBI promoter, resulting in TGFBI downregulation. Moreover, SUV39H2 interacted with Stat1 to transcriptionally downregulate TGFBI. Stat1 is a key regulator in inflammatory diseases. For instance, Stat1‐induced M1 macrophage polarisation contributes to sepsis‐induced MOF [52]. Stat1‐mediated pyroptosis of macrophages promotes the progression of bacterial meningitis [53]. Interestingly, targeting Stat1 inhibited the pyroptosis of macrophages and alleviated septic shock‐induced MOF in septic shock. Therefore, SUV39H2‐Stat1‐mediated inhibition of TGFBI transcription contributed to macrophage pyroptosis and the progression of septic shock.

## 5 Conclusion

In conclusion, transcriptional inhibition of TGFBI induced by SUV39H2‐Stat1 in septic shock mediated the pyroptosis of macrophages, resulting in an inflammatory response and sepsis‐induced MOF. Therefore, TGFBI may be a promising target for septic shock.

## Author Contributions

**Suzhao Zou:** data curation (equal), formal analysis (equal), investigation (equal), methodology (equal), software (equal), supervision (equal), validation (equal), writing – original draft (lead). **Lan Li:** conceptualization (lead), funding acquisition (lead), project administration (lead), writing – original draft (equal). **Bo Lv:** data curation (equal), formal analysis (equal), investigation (equal), methodology (equal), resources (equal), software (equal), supervision (equal), validation (equal), visualization (equal).

## Conflicts of Interest

The authors declare no conflicts of interest.

## Supporting information


**Figure S1.** TGFBI inhibited M1 macrophage polarisation (A, B) The percentages of TNF‐α^+^CD86^+^ cells were detected using flow cytometry. ***p < 0.001.

**Figure S2.** TGFBI promotes the bacterial killing ability of macrophages (A, B) THP‐1 cells were infected with *P. ae* and *E. coli* . TNF‐α^+^CD86^+^ cells were detected using flow cytometry. **p < 0.01, ^#^ *p* < 0.05, ^##^ *p* < 0.01.

**Figure S3.** WED inhibited M1 macrophage polarisation (A, B) The percentages of TNF‐α^+^CD86^+^ cells were detected using flow cytometry. ***p < 0.001.

**Table S1.** The sequences of the siRNAs.

**Table S2.** The sequences of the primers used in PCR.

**Table S3.** Demographic and clinical features of septic shock patients.

## Data Availability

The data generated in the present study may be requested from the corresponding author.
