# Supplementary figures and images for: TGFBI Inhibits the Pyroptosis of Macrophages to Ameliorate Septic Shock
Source: J Cell Mol Med. 2025 Oct 13;29(19):e70802. doi: 10.1111/jcmm.70802 (PMC12516155; doi:10.1111/jcmm.70802)

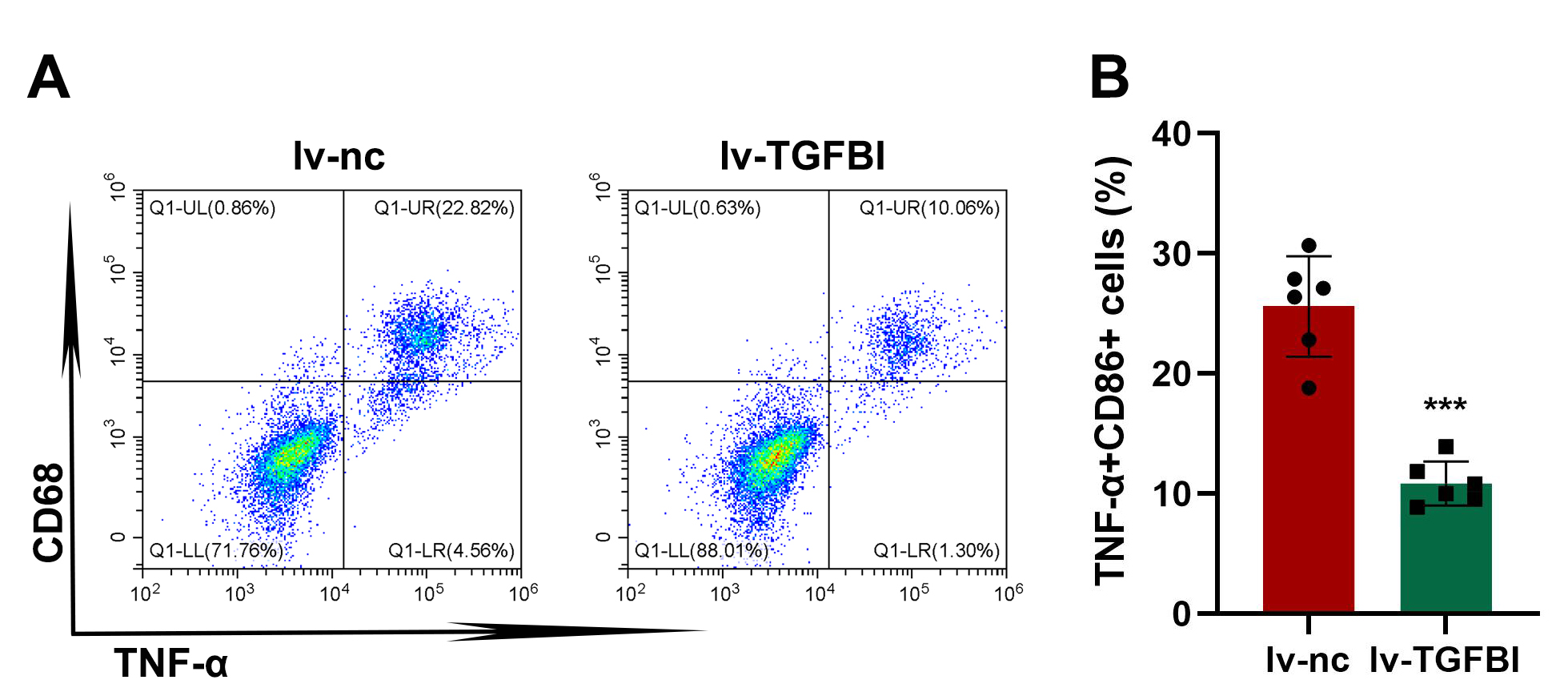

Supplement: Supplementary file 1 — Figure S1. TGFBI inhibited M1 macrophage polarisation (A, B) The percentages of TNF‐α+CD86+ cells were detected using flow cytometry. ***p < 0.001. [file JCMM-29-e70802-s005.jpg]

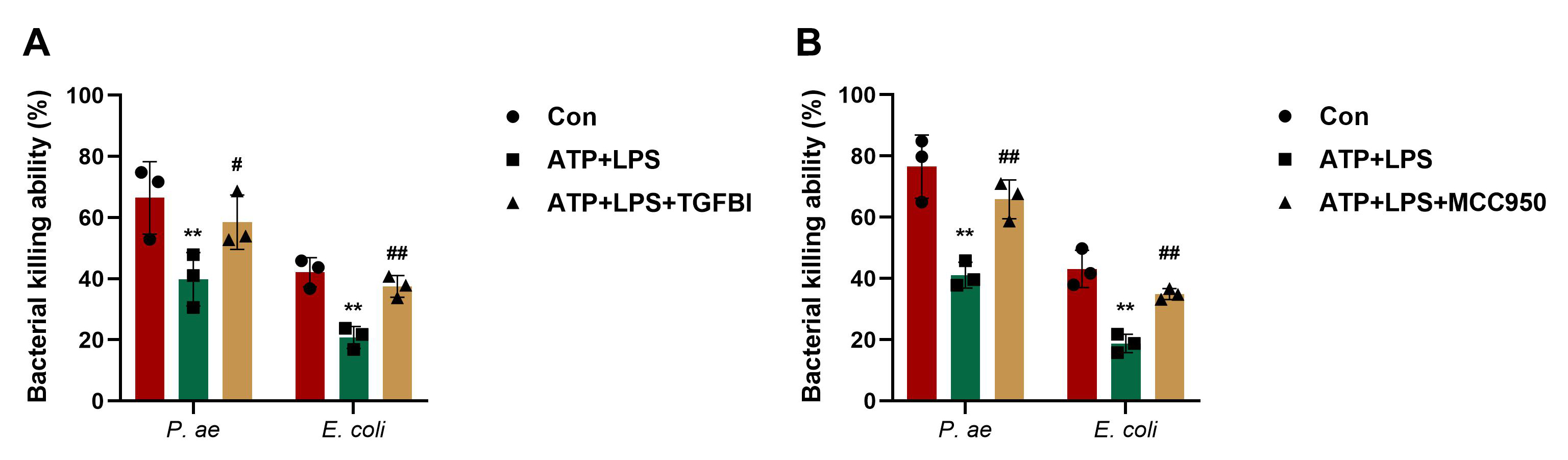

Supplement: Supplementary file 2 — Figure S2. TGFBI promotes the bacterial killing ability of macrophages (A, B) THP‐1 cells were infected with P. ae and E. coli . TNF‐α+CD86+ cells were detected using flow cytometry. **p < 0.01, # p < 0.05, ## p < 0.01. [file JCMM-29-e70802-s006.jpg]

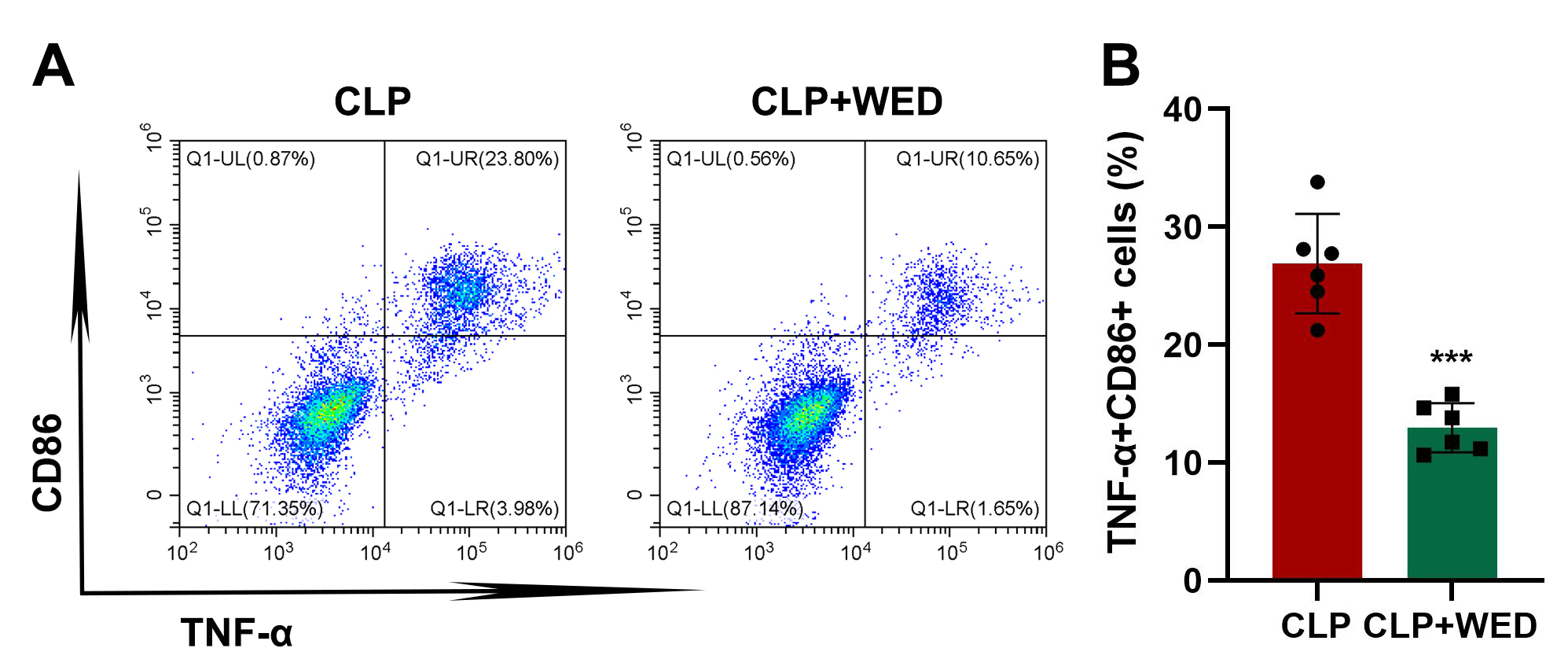

Supplement: Supplementary file 3 — Figure S3. WED inhibited M1 macrophage polarisation (A, B) The percentages of TNF‐α+CD86+ cells were detected using flow cytometry. ***p < 0.001. [file JCMM-29-e70802-s003.jpg]
