# Supplementary material for: TGFBI Inhibits the Pyroptosis of Macrophages to Ameliorate Septic Shock
Source: J Cell Mol Med. 2025 Oct 13;29(19):e70802. doi: 10.1111/jcmm.70802 (PMC12516155; doi:10.1111/jcmm.70802)
Supplement: Supplementary file 4 — Table S1. The sequences of the siRNAs. [file JCMM-29-e70802-s001.docx]

**Supplementary table 1. The sequences of siRNAs**

| **Names** | **Sequences** |
| --- | --- |
| SUV39H2i | F: 5’-GGAGGGGUGCAGUGUCUGGTT-3’ |
|  | R: 5’-CCAGACACUGCACCCCUCCTT-3’ |
| Stat1i | F: 5’-GCAGGUUCACCAGCUUUAUTT-3’ |
|  | R: 5’- AUAAAGCUGGUGAACCUGCTC-3’ |
