# Supplementary material for: TGFBI Inhibits the Pyroptosis of Macrophages to Ameliorate Septic Shock
Source: J Cell Mol Med. 2025 Oct 13;29(19):e70802. doi: 10.1111/jcmm.70802 (PMC12516155; doi:10.1111/jcmm.70802)
Supplement: Supplementary file 5 — Table S2. The sequences of the primers used in PCR. [file JCMM-29-e70802-s002.docx]

**Supplementary table 2. The sequences of the primers used in PCR**

| **Names** | **Sequences** |
| --- | --- |
| NLRP3 | F: 5’-GATCTTCGCTGCGATCAACAG-3’ |
|  | R: 5’-CGTGCATTATCTGAACCCCAC-3’ |
| ASC | F: 5’-TGGATGCTCTGTACGGGAAG-3’ |
|  | R: 5’-CCAGGCTGGTGTGAAACTGAA-3’ |
| GSDMD | F: 5’-GTGTGTCAACCTGTCTATCAAGG-3’ |
|  | R: 5’-CATGGCATCGTAGAAGTGGAAG-3’ |
| GAPDH | F: 5’-TGTGGGCATCAATGGATTTGG-3’ |
|  | R: 5’-ACACCATGTATTCCGGGTCAAT-3’ |
