# Supplementary material for: TGFBI Inhibits the Pyroptosis of Macrophages to Ameliorate Septic Shock
Source: J Cell Mol Med. 2025 Oct 13;29(19):e70802. doi: 10.1111/jcmm.70802 (PMC12516155; doi:10.1111/jcmm.70802)
Supplement: Supplementary file 6 — Table S3. Demographic and clinical features of septic shock patients. [file JCMM-29-e70802-s004.docx]

**Supplementary Table 3. Demographic and clinical features of septic shock patients.**

| **Main parameters** | **Patients (n = 66)** | **Volunteers (n = 22)** |
| --- | --- | --- |
| **Age (years)** | 48.21±10.8 | 48.93±11.43 |
| **Gender (male)** | 52 | 17 |
| **Mechanical ventilation (n)** | 28 | NA |
| **Antibiotic administration (n)** | 37 | NA |
| **Surgery (number of interventions)** | 37 | NA |
| **White blood cell count (×10^9^/L)** | 16.3±3.6^a^ | 5.8±2.4 |
| **Lymphocyte count (×10^9^/L)** | 0.87±0.08^a^ | 1.64±0.58 |
| **BNP (pg/L)** | 367.75±105.5^a^ | 22.46±2.17 |
| **CRP (mg/L)** | 146.35 ± 7.83^a^ | 7.58 ± 1.91 |
| **PCT (ng/L)** | 12.88 ± 3.57^a^ | 0.17 ± 0.03 |
| **APACHE score** | 26 ± 4 | NA |
| **SAPS score** | 54 ± 4 | NA |

^a^*P* < 0.05 was considered statistically significant. Data were presented as the mean ± SD. BNP, Brain Natriuretic Peptide. CRP, C-type Reactive Protein. PCT, procalcitonin. APACHE, Acute Physiology and Chronic Health Evaluation; NA, not applicable; SAPS, Simplified Acute Physiology Score.
